# Optimal Design of Electromagnetically Actuated MEMS Cantilevers

**DOI:** 10.3390/s18082533

**Published:** 2018-08-02

**Authors:** Paolo Di Barba, Teodor Gotszalk, Wojciech Majstrzyk, Maria Evelina Mognaschi, Karolina Orłowska, Sławomir Wiak, Andrzej Sierakowski

**Affiliations:** 1 Department of Electrical, Computer and Biomedical Engineering, University of Pavia, I-27100 Pavia, Italy; paolo.dibarba@unipv.it; 2 Faculty of Microsystem, Electronics and Photonics, Wrocław University of Science and Technology, PL-50372 Wrocław, Poland; teodor.gotszalk@pwr.edu.pl (T.G.); wojciech.majstrzyk@pwr.edu.pl (W.M.); karolina.orlowska@pwr.edu.pl (K.O.); 3 Institute of Mechatronics and Information Systems, Łódź University of Technology, PL-90924 Łódź, Poland; slawomir.wiak@p.lodz.pl; 4 Institute of Electron Technology, PL-02668 Warsaw, Poland; asierak@ite.waw.pl

**Keywords:** electromagnetically actuated cantilevers, nanometrology, multiobjective optimization, active cantilevers, SOI-based prototyping

## Abstract

In this paper we present the numerical and experimental results of a design optimization of electromagnetic cantilevers. In particular, a cost-effective technique of evolutionary computing enabling the simultaneous minimization of multiple criteria is applied. A set of optimal solutions are subsequently fabricated and measured. The designed cantilevers are fabricated in arrays, which makes the comparison and measurements of the sensor properties reliable. The microfabrication process, based on the silicon on insulator (SOI) technology, is proposed in order to minimize parasitic phenomena and enable efficient electromagnetic actuation. Measurements on the fabricated prototypes assessed the proposed methodological approach.

## 1. Introduction

Micro-electromechanical systems (MEMS) are micromachines containing movable parts whose deflection is controlled and detected electronically. In general MEMS are manufactured using technologies applied in microelectronics. In this way fabrication of devices of various functions and properties can be done in a batch process, which increases repeatability and system reliability. 

The critical physical dimensions of MEMS devices, to which belong simple structures having no moving elements and extremely complex electromechanical systems with elements actuated and controlled by the integrated microelectronics, can vary from several microns to several millimeters. In a natural way the MEMS technology merges at the nanoscale into nano-electromechanical systems (NEMS). Among others, the so called supported cantilevers, whose elasticity can be described with relatively high accuracy using simple models, are MEMS devices. Moreover, the variety of possible applications is very broad, ranging from scanning probe microscopy (SPM) to sensing systems applied in biochemistry and biotechnology [1]. In all the aforementioned applications the cantilever static or resonance deflection is detected to follow the phenomena of interest. Optical and electrical techniques, which can be applied in single or array cantilever (sensor) operation, enable monitoring of thermomechanical noise structure vibration. However, it should be mentioned that the full interoperability of the cantilever system can only be obtained when the structure deflection is electrically controlled. This means that the deflection should be maintained in the feedback loop by a deflection actuator, which is integrated with the movable mechanical part. This way, as the mass of the cantilever is quite small, the actuator induces device movement with the highest energetic efficiency and speed. Moreover, the actuation reliability is improved as the deflection is actuated of only the movable part. There are various electrical technologies for actuation of the cantilever displacement. The application of electrostatic actuation scheme is usually limited to the cantilevers of big dimensions [2]. Moreover, it involves electrode biasing with relatively high voltage which cannot often be applied in MEMS technology. 

The cantilever deflection can be controlled piezoelectrically as well [3]. In this solution a piezoelectrical thin film is deposited on the cantilever and when it is electrically biased the structure displacement can be excited. Similar to the electrostatic technology, the actuator bias voltage is quite high which hinders the applications in the integrated sensing systems. Moreover, deposition of the piezoelectric thin film is cumbersome and its repeatability is often limited. From the fabrication point of view, the electrothermal actuator technology is significantly simpler [4]. In this case, a spring beam integrates a microheater dissipating heat due to Joule effect. As a consequence of different thermal expansion coefficients of materials, forming the entire structure, mechanical stress occurs in the movable part [5]. As a result the structure deflects. In this way the deflection in the frequency range of up to several MHz can be induced [6]. In the DC regime the cantilever can be deflected by even few micrometers [4]. The drawback of the electrothermal actuation scheme is that its application in liquids and consequently in biochemistry and biotechnology is limited. In this case, the heat generated in the microheater can not only propagate through the beam structure itself, but also in the surrounding liquid, reducing significantly the actuation efficiency. Moreover, in the electrothermal scheme the cantilever deflection can be controlled in one direction only.

In contrast to that, the electromagnetic actuation technology is free from aforementioned drawbacks. In this approach the cantilever integrates a conductive loop called Lorentz loop. When the electromagnetic cantilever is electrically biased and immersed in the magnetic field, the electromagnetic force induces deflection [6,7].

The bidirectional cantilever deflection can be analysed based on the model describing the electromagnetic force in Lorentz loop. The actuation force can be computed by means of the Lorentz force equation. The strong magnetic field in the range of fractions of a tesla can be excited by external magnets. The current in the loop can be controlled by the low voltage electronics and the length of Lorentz loop can be determined with high accuracy. In this way DC and AC displacement can be controlled with the highest precision, efficiency and reliability. Moreover, it is relatively straight forward to design systems operating at very low energy integrated with Application Specific Integrated Circuits (ASICs). The electromagnetic cantilever was introduced for the first time by Shen [8]. At the resonance, the cantilevers can be observed with high resolution, hence they were also applied as high resolution magnetic field sensors [9,10] and resonators [11].

The actuation precision and reliability were the main reasons, why the electromagnetic cantilevers were successfully applied in SPM [12,13]. In all these investigations the cantilever deflection was determined metrologically (in other words quantitatively), which is of significant importance for investigations of the interactions at a scanning probe microscopy tip. 

The electromagnetic cantilevers were also utilized in metrology of the mechanical stress associated with the adsorbtion of molecular self-assembled layers (SAMs) on the gold cantilever surface. When thiol molecules adsorb (covalently bind) on the cantilever surface, mechanical stress occurs between molecular film and the cantilever leading to the structure deflection. Such a response was usually used to detect (indicate qualitatively) chemical surface reactions with high resolution [14]. 

In our experiments the cantilever deflection caused by the molecular adsorbtion was observed optically and the electromagnetic force was applied to compensate the structure deflection. As the electromagnetic force was determined precisely by the bias current, the magnetic field and the Lorentz loop dimensions, the electro-mechanical phenomena were described quantitatively for the first time [15]. 

Despite the many advantages of electromagnetic actuation, its limitations must be identified as well. To the most important problems, which must be taken into account, belongs parasitic thermomechanical structure actuation when Lorentz loop is electrically biased. In this case, due to various coefficients of linear extension of the materials forming the spring beam, heat dissipated in the structure leads to additional structure deflection. 

The spring beam should exhibit low stiffness in order to control the structure deflection in the range of up to several micrometers, which additionally correlates with higher force and mass change detection resolution. Moreover, the resonance frequency of the designed and fabricated electromagnetic structures must be as high as possible, which makes it insensitive to the measurement disturbances and decreases the time response. The low stiffness and high resonance frequency can be achieved when the length and the thickness of the cantilever are reduced in the appropriate way. However, in the case of the electromagnetic cantilevers one should also optimize the structure geometry in order to reduce Lorentz loop resistance. In this way the heat dissipated in the biased beam and the parasitic thermomechanical actuation are significantly reduced. Unfortunately, simple analysis of the equations modelling the electromagnetic cantilevers does not allow a closed-form design solution and only the optimal design methods make it possible to overcome the abovementioned limitations. In general, the methods of automated optimal design are based on repeated analysis to solve the field model [16], which ultimately influence the computational budget of the simulation. 

Against this background, the available technology makes it possible to fabricate electromagnetically actuated cantilevers with no severe limitation on the shape. On the other hand, high accuracy measurements demand low stiffness, low power losses and high resonance frequency. Hence, the exploitation of methods for automated optimal design help to achieve these requirements with subsequent prototyping of better performing cantilever. A contribution towards this goal is here presented. Specifically, the paper is organized as follows: in Section 2.1 and Section 2.2 forward and inverse models describing the behavior of the cantilever are presented. In Section 2.3 the fabrication process is described. In Section 3.1, Section 3.2 and Section 3.3 the optimization results are presented, while in Section 3.4 and Section 3.5 the measurement results are shown. Finally, a discussion is presented and conclusions are drawn.

## 2. Materials and Methods

**Figure 1:**
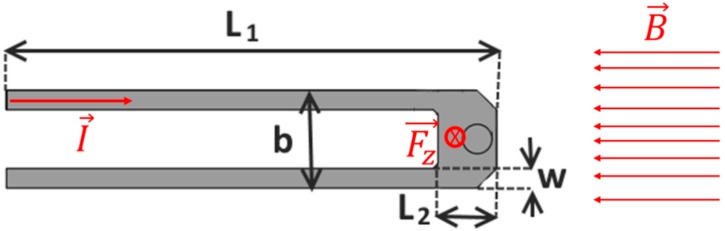
Design variables of the cantilever (black symbols) and schematic representation of the electromagnetic actuation by means of the Lorentz force (red lines).

The deflection of the MEMS device can be induced in the electromagnetic way. In this technology external magnetic field interacts with the current flowing through conductive loop (Figure 1) producing a Lorentz force **F_z_** [6,7].

### 2.1. Optimal Design of an Electromagnetically Actuated Cantilever: Direct Problem

The direct (or analysis) problem reads as follows given the shape **g** of the cantilever end, current **I**, and magnetic induction **B**, find:

the stiffness *k* of the cantilever;
the resonance frequency *f* of the cantilever;
the force *F_z_* acting on the end region and its displacement Δ*z*;
the electric resistance *R* (power-loss related) of the Lorentz loop.

The stiffness *k* of the cantilever can be calculated [17] as follows:

(1) $$k=\frac{Ebwt^{3}}{2b\left(L_{1}^{3}-L_{2}^{3}\right)+4wL_{2}^{3}}\;\left[{\mathrm{Nm}}^{-1}\right],$$

where *E* is the Young’s modulus, *b* is the cantilever width, *w* the arm width, *t* is the thickness equal to 1.5 µm, *L*_1_ is the cantilever length and *L*_2_ is the tip length (see Figure 1).

The resonance frequency *f* can be evaluated with the following approximate formula [18]:

(2) $$f≅0.161\frac{t}{L_{1}^{2}}\sqrt{\frac{E}{\rho }}\;\left[\mathrm{rad}\;s^{-1}\right],$$

where *ρ* is the mass density equal to 2330 kgm^−3^.

The force *F_z_* and its displacement Δ*z* can be calculated as follows:

(3) $$F_{z}=IbB,$$

(4) $$∆z=\frac{F_{z}}{k}.$$

Equation (3), which is derived from the Lorentz’s equation, is under the assumption that the cantilever, i.e., the plane in which the current flows, is perpendicular to the magnetic induction field.

Finally, the electric resistance *R* can be calculated as the series of three electric resistances of the three path components (two arms, with the same resistance value *R*_1_ and the tip, with resistance *R*_2_):

(5) $$R=2R_{1}+R_{2}≅2\frac{\sigma^{-1}\left(L_{1}-L_{2}\right)}{wt}+\frac{\sigma^{-1}b}{L_{2}t},$$

where σ is the electric conductivity of the boron-doped silicon (without metal layer) equal to 6.67 × 10^4^ Sm^−1^.

### 2.2. Optimal Design of an Electromagnetically Actuated Cantilever: Inverse Problem

If the shape of the cantilever end is defined by means of a *n*-dimensional vector ***g*** = (*g*_1_, …, *g_k_*, …, *g_n_*) of geometric variables (e.g., for a polygonally-shaped end region, the coordinates of the relevant vertices), the inverse (or design) problem reads: given current *I* and magnetic induction *B*, find the shape ***g*** = (*g*_1_, …, *g_k_*, …, *g_n_*) of the cantilever end region such that:

the stiffness *k*(***g***) of the cantilever is minimized;
the resonance frequency *f*(***g***) is maximized;
the displacement Δ*z*(***g***) of the end region is maximized;
the electric resistance *R*(***g***) of the Lorentz loop is minimized.

A multi-objective optimization problem characterized by four objective functions [*k*(***g***), *f*(***g***), Δ*z*(***g***), *R*(***g***)] is originated. When more than one objective function is considered in the optimization, more solutions, belonging to the so-called Pareto front, are obtained. In particular, a solution is called Pareto optimal if there does not exist another solution that dominates it i.e., a solution that cannot be improved in any of the objectives without degrading at least one of the other objectives.

Considering *n*-objective functions, a solution ***g*_1_** is said to dominate another solution ***g*_2_**, if:

(6) $$f_{i}\left(g_{1}\right)\leq f_{i}\left(g_{2}\right)\;\;\forall \;i\in \left(1,n\right)\;\;and$$

(7) $$\exists \;j\;\in \;\left(1,n\right)\;such\;that\;f_{j}\left(g_{1}\right)<f_{j}\left(g_{2}\right)\;.$$

A solution ***g*_1_** is called Pareto indifferent with respect to a solution ***g*_2_** if:

(8) $$\exists \;j\;\in \;\left(1,n\right)\;such\;that\;f_{j}\left(g_{1}\right)<f_{j}\left(g_{2}\right)\;and$$

(9) $$f_{i}\left(g_{1}\right)\leq f_{i}\left(g_{2}\right)\;\;\forall \;i\neq j\;\in \;\left(1,n\right)$$

In our inverse problem it turns out to be *n* = 4 and *f*_1_ = *k*(***g***), *f*_2_ = *f*(***g***), *f*_3_ = Δ*z* (***g***) and *f*_4_ = *R*(***g***). Our goal is, starting from a prototype geometry ***g*_0_**, to find a new geometry improving ***g*_0_** against the four objectives, according to Equations (6)–(9).

When many objective functions (say more than two) are considered, it is very common to find solutions of the optimization problem which are indifferent in the Pareto sense to the starting point. However, these solutions are nevertheless interesting because they improve at least one objective function.

It can be noted that Equations (1)–(5) define an analytical model for the direct problem, however, Equations (6)–(9) prevent from an analytical solution of the inverse problem and therefore the numerical method is in order.

The shape of the cantilever is defined by four design variables, as shown in Figure 1:

*w*, arm width.
*L*_1_, cantilever length.
*L*_2_, tip length.
*b*, cantilever width.

The variation range for each design variable is shown in Table 1. The chosen values for the boundaries are based on the experience.

In order to guarantee a geometrical congruency, the following constraint (units in µm) is set:

(10) b≥2w+10.

A series of optimizations are subsequently run, considering one (Opt1), two (Opt2) or three (Opt3) objective functions at a time:

Opt1—each objective function i.e., *k*, *f*, Δ*z* and *R*, is individually optimized in four different single-objective optimizations (Opt1k, Opt1f, Opt1z, Opt1R). In each case, the activated objective function is the leading one, while the remaining three objective functions are updated, depending on the current value of the design vector g;

Opt2—the following optimizations are run: *k* and *R* are optimized (Opt2kR), *f* and *z* are optimized (Opt2fz), *f* and *R* are optimized (Opt2fR), *z* and *R* are optimized (Opt2zR). Each pair of objective function is optimized in the Pareto sense, the remaining two functions are updated, depending on the current value of the design vector ***g***;

Opt3—*k*, *z* and *R* are optimized (Opt3) in the Pareto sense, while the frequency *f*(***g***) is simply updated.

In order to solve these optimization problems, an evolutionary algorithm of lowest order is applied [19]. This algorithm is able to solve single-objective problems (in this case it is called “ESTRA method” [20,21]) and multi-objective problems (“MOESTRA method” [22]). The search in the design space begins in a region of radius **d_0_** (standard deviation) centered at the initial point **m_0_** (mean value); **m_0_** is externally provided, while **d_0_** is internally calculated on the basis of the bounds boxing the variation of the design variables.

Setting **m** = **m_0_** and **d** = **d_0_**, the *generation* of the design vector **x** = **m** + **u d** then proceeds, resorting to a normal sample u∈(0,1). It is verified that ***x*** fulfils bounds and constraints (i.e., that ***x*** is feasible), otherwise a new design vector is generated until it falls inside the feasible region.

The associated objective function *f*(**x**) is then evaluated and the test if *f*(**x**) dominates *f*(**m**) (Equations (6) and (7)) is performed; if the test is successful, **m** is replaced by **x** (the so-called *selection* process), otherwise **m** is retained.

The next step is concerned with the size of the search region that will be used for the successive iterations. The underlying rationale is that when a point better than the current one is found, the radius of the search region is increased around the new point to search for further improvements; if no improvement is found, the radius of the search region is gradually decreased up to convergence (*annealing* process). 

In this respect, the evolutionary algorithm substantially differs from a deterministic one e.g., Nelder and Mead algorithm [23], in which the search region would be narrowed around the better point in order to converge towards the corresponding, nearest minimum. The drawback is that this minimum might be a local one. On the contrary, the evolutionary algorithm, if successful in finding a better point, covers a larger region of search in order to see if there would be another good candidate in the neighborhood, and then does the opposite when this is not deemed possible. This way, there is a non-zero probability of finding the region where the global optimum of the objective function is located. To assess the optimization results, a set of prototypes has been fabricated based on the technology described in the subsequent Section.

### 2.3. Fabrication Process

The fabrication process of the microcantilevers used for a radiation pressure sensing was based on a double side micromachining concept [24]. However, in contrast to the typical technology based on bulk silicon substrates, in this case the silicon on insulator (SOI) wafers with 1 and 1.5 micrometers thick buried oxide and cantilever layer, respectively, were used as the input material. Despite the fact that the use of SOI substrates is more expensive, this solution has many advantages compared to the use of the bulk wafers. Two advantages of using the SOI substrates are particularly important.

The first advantage is a significant simplification of the microcantilever production process. The second advantage is a guarantee that all cantilevers defined on one wafer are characterized by uniform thickness regardless of its shape and size (the thickness depends only on the SOI wafer cantilever layer properties).

**Figure 2:**
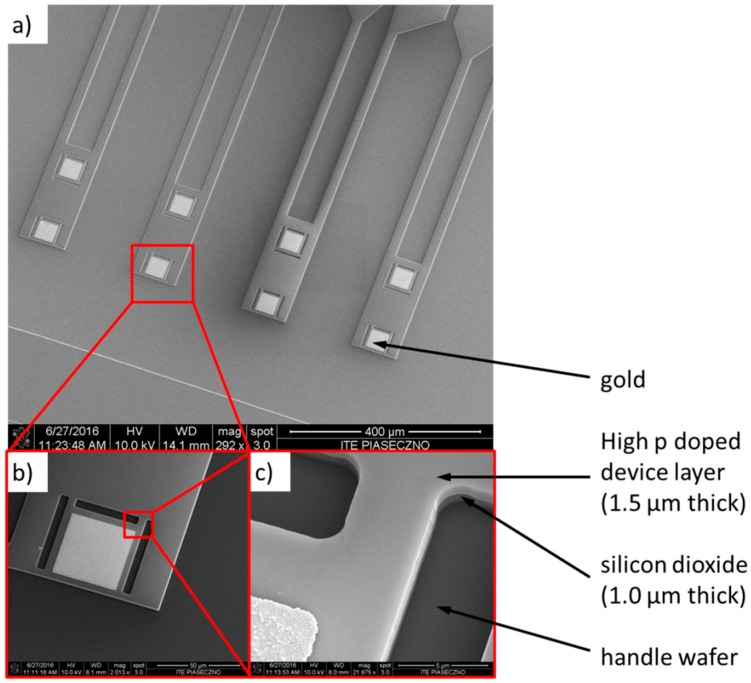
The scanning electron microscope (SEM) image of the cantilever matrix after three technological steps (inter-operative control).

**Figure 3:**
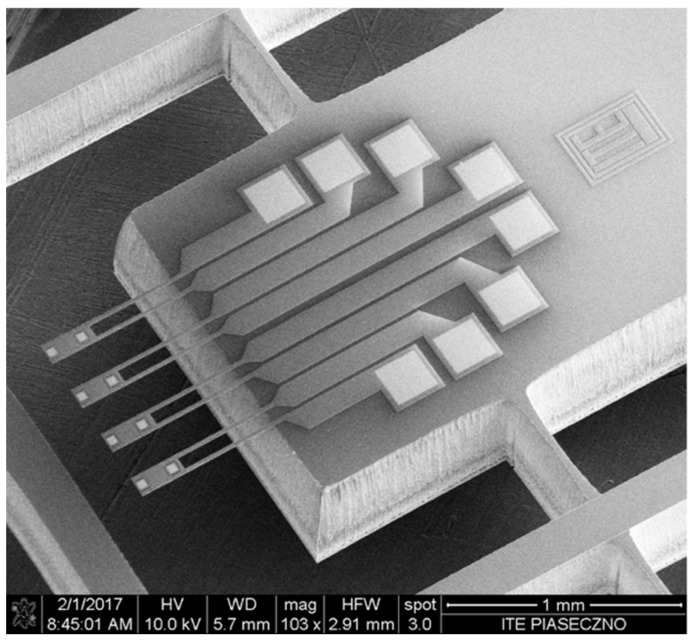
The scanning electron microscope (SEM) image of the final structure. Scanning parameters of the image. High Voltage (HV) = 10 kV; Working Distance (WD) = 5.7 mm; Horizontal Field of View (HFW) = 2.91 mm.

Therefore, using the SOI substrate, the production technology consists of only four technological steps: high p doping of the whole cantilever layer, definition of a gold contacts and mirrors, definition of the shape of the cantilever and finally the releasing of the cantilevers. Figure 2 presents the scanning electron microscopy (SEM) image of the cantilever after three steps, i.e., after plasma etching processes. Upon magnification (Figure 2b,c) the four layers can be observed: gold (which serves as a mirror), a 1.5 μm silicon layer, a buried silicon dioxide and a handle silicon wafer. The example final cantilevers matrix after released operation is presented in Figure 3.

The presented construction was optimized for the electromagnetic actuation, first proposed by Buguin group [25]. The use of SOI wafer makes it easy to obtain a homogeneous doping of the cantilever defined in the SOI cantilever layer, which significantly reduced the thermal actuation effect.

The homogeneous high-p-doped cantilever layer was obtained by using boron doped layer deposited by Low Pressure Chemical Vapor Deposition (LPCVD) method [26]. In this method there are two technological stages: boron source layer deposition using B_2_H_6_ 5% in N_2_ at 270 °C (pre-diffusion) and high temperature annealing (diffusion of the boron dopant from previously deposited layer into silicon). The boron dopant profile can be optimized by controlling the duration time of pre-diffusion and duration time and temperature of the diffusion step.

By changing the pre-diffusion time we can control the surface boron concentration. In our experiments, the duration of the pre-diffusion was 55 min. Relatively long times allow one to obtain a high boron concentration of 1 × 10^20^ cm^−3^.

On the other hand, changing the temperature and duration time of diffusion allows one to control the final dopant profile in the device layer. In our case the temperature and duration time of the diffusion were 1100 °C and 25 min, respectively. Further increases in value of these parameters would cause an undesirable effect of reducing the surface boron concentration. In this case the dopant profile would not be homogeneous and would increase the thermal actuation effect. On the other hand, decreasing the diffusion temperature causes incomplete oxidation of the layer used as a source of boron, increasing its etch resistance, and makes its removal difficult.

**Figure 4:**
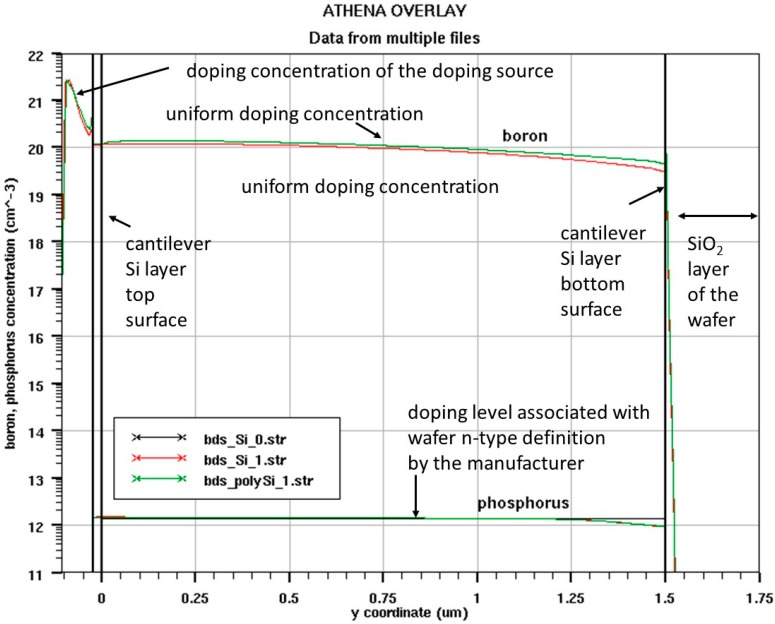
The high concentration of p dopant layer simulation result for the silicon on insulator (SOI) cantilever. Red and green curves represent two methods of doping concentration calculation.

The results of simulation of the obtained dopant profile for the listed above process parameters are presented in Figure 4. The simulation confirmed that the boron dopant profile is uniform along the cantilever layer thickness; thus, the thermal expansion coefficient should be also constant [27].

## 3. Results

### 3.1. Single-Objective Optimization Results

The results of the single-objective optimizations are shown in Table 2. In each table the values of the design variables and of the functions (*k*, *f*, Δ*z*, *R*) are shown. In particular, the minimized objective function is highlighted in bold.

From Table 2—Opt1k, it can be noted that the non-controlled objective functions *f* decreases, which is undesirable, Δ*z* increases (desirable) and *R* increases (undesirable).

From Table 2—Opt1f, it can be noted that the non-controlled objective functions *k* increases, which is undesirable, Δ*z* decreases (undesirable) and *R* decreases (desirable).

From Table 2—Opt1z, it can be noted that the non-controlled objective functions *k* decreases (desirable), *f* decreases (undesirable) and *R* increases (undesirable).

From Table 2—Opt1R, it can be noted that the non-controlled objective functions *k* increases (undesirable), *f* increases (desirable) and Δ*z* decreases (undesirable).

### 3.2. Bi-Objective Optimization Results

The results of the bi-objective optimizations are shown in Table 3. In each table the values of the design variables and of the functions (*k*, *f*, Δ*z*, *R*) are shown. In particular, the minimized objective functions are highlighted in bold.

From Table 3—Opt2kR, it can be noted that the non-controlled objective function *f* decreases, which is undesirable, but Δ*z* increases (desirable).

From Table 3—Opt2fz, it can be noted that the non-controlled objective function *k* increases, which is undesirable, but *R* decreases (desirable).

From Table 3—Opt2fR, it can be noted that the non-controlled objective function *k* increases (undesirable) and Δ*z* decreases (undesirable).

From Table 3—Opt2zR, it can be noted that the non-controlled objective function *k* decreases (desirable) and *f* decreases (undesirable).

### 3.3. Tri-Objective Optimization Results

The results of the tri-objective optimization are shown in Table 4. The values of the design variables and of the functions (*k*, *f*, Δ*z*, *R*) are shown. In particular, the minimized objective functions are highlighted in bold.

From Table 4, it can be noted that the non-controlled objective function *f* decreases, which is undesirable. The results obtained with the tri-objective optimization are the most interesting ones, at least from the methodological viewpoint. However, we note that, depending on the designer’s purpose and preferences, the results obtained with one or two objectives (Table 2 and Table 3, respectively) could be preferred than the ones obtained with three objectives (Table 4). In general, in fact, the improvements achieved by means of a single- or bi-objective optimization could be substantial, even if the non-controlled objectives might undergo a modest deterioration. This is sometimes the case in engineering practice.

From the practical viewpoint, the solutions found in Opt2fz and Opt3 are felt to be the best ones, as explained in the following Section 3.2. Hence these cantilevers, as well as the initial cantilever, were fabricated and measured to verify the design and assess the optimization results.

### 3.4. Measurements on Optimal Cantilevers

**Figure 5:**
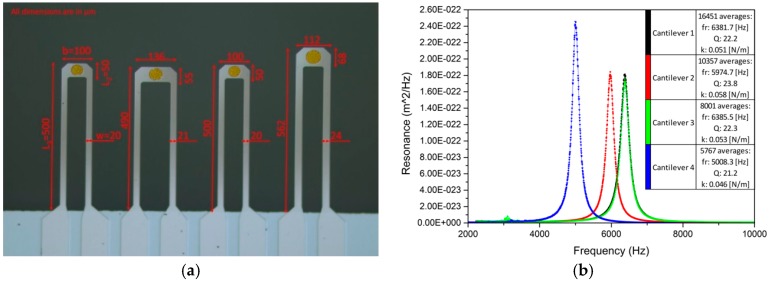
(**a**) example of manufactured cantilever array consisting of four cantilevers. Cantilevers 1 and 3 (from left to right) correspond to the initial design, while 2 and 4 are the optimal cantilevers after optimizations Opt2fz and Opt3. (**b**) the resonance response of the manufactured cantilevers.

The cantilevers according to the initial design as well as of the shapes obtained from Opt2fz and Opt3 optimizations were considered to be optimal. With Opt2fz solution, significant increase in deflection and small decrease in resistance of loop were obtained. These two changes were most desirable and came without deterioration in the resonant frequency. Another solution considered to be optimal, the Opt3, exhibits even higher deflection and smaller resistance at the cost of 15% lower frequency. These two cantilevers were fabricated and finally measured. The example array is shown in Figure 5a. This manufactured cantilever array consists of four cantilevers, in particular:

- two cantilevers corresponding to initial design,
- final design, according to Opt2fz optimization result
- final design, according to Opt3 optimization result

To verify their properties, we have measured the thermomechanical noise of the cantilevers. The power spectrum of each resonant mode in the thermomechanical noise is defined by the following equation [28]:

(11) $$X_{th}\left(f\right)=2k_{B}T\pi^{-1}Q^{-1}f_{0}^{3}k^{-1}{\left[{\left(f_{0}^{2}-f^{2}\right)}^{2}+f_{0}^{2}f^{2}Q^{-2}\right]}^{-1},$$

where *Q* is the quality factor, *k_B_* is the Boltzmann constant, *T* is the absolute temperature. By fitting parameters of Equation (11) to the measurements [18], the mechanical parameters are obtained. The thermomechanical noise formula is a result of the equipartition theorem [29]. Due to this theorem an object, which dissipates energy in thermal equilibrium, is subject to a fluctuation force. The measured resonance responses of the cantilevers shown in Figure 5a are shown in Figure 5b.

Two cantilever arrays, each of which containing twice the same initial design cantilever, were fabricated. The measurement was then performed for both cantilever arrays, hence for four cantilevers (Table 5). This measurement provides information about the cantilever resonant frequency and its stiffness. Additionally, the resistance of the loop was measured. The cantilever provides direct electrical contacts. A Keithley 2000 multimeter was used to measure the resistance of the loop. The measurement was conducted in DC mode. The Keithley multimeter applies a 100 µA current and subsequently the voltage drop was measured. The results of manufactured cantilever arrays according to initial design are shown in Table 5. The measured parameters of the 8 cantilevers of initial design vary at most by 8% from the mean values.

The cantilever parameters manufactured according to Opt2fz and Opt3 optimization results are provided in Table 6.

### 3.5. Electromagnetic Actuation of the Cantilevers

**Figure 6:**
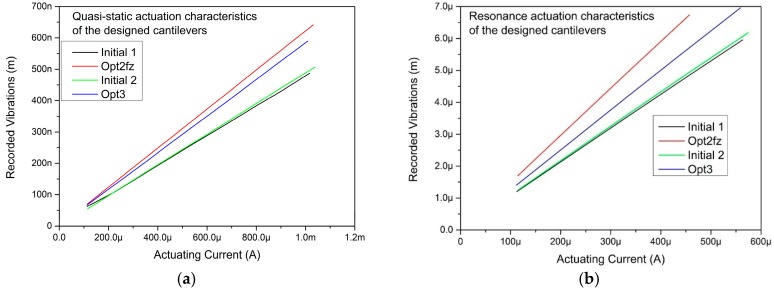
Results of the electromagnetic actuation of the optimal cantilevers (**a**) results recorded in the low frequency region (100–1000 Hz) and (**b**) in resonance. The experiments were performed under uniform 317 mT magnetic field.

One of the manufactured arrays was subject to electromagnetic actuation. The array was placed in a uniform magnetic field of 317 mT. Then the amplitude of vibration vs actuating current characteristics were recorded. Such experiment was performed for each cantilever in the array. During the experiment the current was flowing only through one of the cantilevers at a time. Figure 6 contains the results of the electromagnetic actuation characterisation. The actuation was performed in resonance and in low frequency region (100–1000 Hz)—so called quasi static characteristics. It can be noted that under the same conditions, the manufactured cantilevers of OPT2fz and OPt3 design exhibit significantly higher vibration amplitudes Δ*z*.

## 4. Discussion

The measured results of the initial cantilever differ slightly from those calculated for the initial prototype in Table 2, Table 3 and Table 4. In fact, the manufactured cantilevers contain the gold layer at each end. This gold is supposed to improve reflection of the laser beam from cantilever surface in AFM measurements. The resonant frequency differs due to the mass of the added gold layer. The resulting frequency of the manufactured cantilevers is lower and equal to approximately 6.5 kHz. It should be also noted that, due to the many technological processes required to manufacture the cantilevers, some drifts in measured parameters are unavoidable. This can be particularly seen for the initial cantilever, where we can compare results for four manufactured pieces and the parameters of each of them differ.

The optimization procedure could account for the mass of the gold layer at the cantilever end. In fact, the gold layer is contained within small area at the end and therefore, it can be treated as a point mass load. This leads to a possibility for the correction of the resonant frequency, using the formula:

(12) $$f=\frac{\sqrt{k}}{m_{e}+m_{g}},$$

where *m_e_* is the effective mass of the cantilever, *m_g_* is the mass of gold. The mass of the gold layer could be assessed from the volume.

The main difference between the optimization results and manufactured cantilevers is the value of the resistance. It comes out from the fact that the value of doping concentration in the manufactured cantilevers is lower than the assumed one in the optimization procedure. However, this discrepancy does not affect the optimization results and its assessment (as it only influences material properties).

Provided that all the optimization runs start from the same initial point, the following remarks can be put forward:

The solutions obtained in Opt1k, Opt1f, Opt1z and Opt1R differ in both design vector (*w*, *L*_1_, *L*_2_, *b*) and objective vector (*k*, *f*, Δ*z*, *R*). This proves that a single solution simultaneously satisfying all the design criteria does not exist. From the optimization theory viewpoint, it is a design conflict problem that can be studied via Pareto optimality.

Solutions obtained in Opt2 and Opt3 can be considered Pareto-equivalent to the initial one, because three objectives improve, while one objective deteriorates (e.g., solution of Opt3 where *k*, Δ*z* and *R* improve, while *f* deteriorates). From the application viewpoint, provided the amount of deterioration in one objective is acceptable, Pareto-equivalent solutions may represent a good alternative to the initial solution.

From the methodological point of view, it can be stated that the applied evolutionary algorithm is suitable for solving the shape optimization of cantilevers. In particular, it can be stated that this method works well in finding an improved cantilever-based device, when dealing with design problems characterized by less than ten design variables and up to three objective functions. In general, this method can be used for a class of optimal shape design problems exhibiting the same complexity in terms of number of design variables and objective functions.

## 5. Conclusions

A class of electromagnetically actuated cantilevers has been considered. Their optimal shape design has been carried out by means of a multi-objective design method, based on evolutionary algorithm. The subsequent fabrication of cantilever arrays and relevant measurements have assessed the optimization results. This puts the ground for a more general procedure of cantilever design for nanometrology purposes.

## Figures and Tables

**Table 1:** Variation range for the design variables (units in µm).

	*w*	*L*_1_	*L*_1_	*b*
Lower bound	20	100	50	100
Upper bound	-	600	100	150

**Table 2:** Single-objective optimization Opt1k results.

	*w* [µm]	*L*_1_ [µm]	*L*_2_ [µm]	*b* [µm]	*k* [Nm^−1^]	*f* [kHz]	Δ*z* [nm]	*R* [Ω]
Initial	20.0	500	50.0	100	4.32 × 10^−2^	8.00	925	470
Opt1k	21.1	569	53.0	114	**3.09 × 10^−2^**	6.18	1482	512
Opt1f	24.6	210	64.6	123	0.726	**45.3**	67.6	138
Opt1z	22.0	568	63.9	128	3.25 × 10^−2^	6.21	**1574**	478
Opt1R	47.6	211	80.7	119	1.39	45.2	34.3	**69.3**

**Table 3:** Bi-objective optimization results.

	*w* [µm]	*L*_1_ [µm]	*L*_2_ [µm]	*b* [µm]	*k* [Nm^−1^]	*f* [kHz]	Δ*z* [nm]	*R* [Ω]
Initial	20.0	500	50.0	100	4.32 × 10^−2^	8.00	925	470
Opt2kR	24.1	557	62.3	110	**3.76 × 10^−2^**	6.45	1171	**429**
Opt2fz	21.0	490	55.4	136	4.82 × 10^−2^	**8.33**	**1129**	438
Opt2fR	60.9	210	86.1	135	1.79	**45.4**	30.3	**56.4**
Opt2zR	27.2	569	57.7	111	3.99 × 10^−2^	6.18	**1113**	**395**

**Table 4:** Tri-objective optimization Opt3 results.

	*w* [µm]	*L*_1_ [µm]	*L*_2_ [µm]	*b* [µm]	*k* [Nm^−1^]	*f* [kHz]	Δ*z* [nm]	*R* [Ω]
Initial	20.0	500	50.0	100	**4.32 × 10^−2^**	8.00	**925**	**470**
Final	24.0	562	68.1	112	**3.65 × 10^−2^**	6.33	**1226**	**429**

**Table 5:** Measurements on the initial cantilever.

Measured Quantities			Computed Quantities			
*R* [kΩ]	*f* [kHz]	*Q*	*k* [Nm^−1^]	*F_min_* [pN]	*I_min_* [nA]	*P_min_* [fW]
1.89	6382	22.2	0.051	0.308	9.63	175
2.26	6386	22.3	0.053	0.313	9.79	217
1.88	6633	23.4	0.060	0.319	9.98	187
2.26	6634	23.4	0.055	0.306	9.55	206
1.88	6461	22.9	0.057	0.319	9.96	187
2.26	6502	22.9	0.056	0.315	9.84	219
1.88	6787	23.9	0.060	0.312	9.76	179
2.26	6799	24.6	0.064	0.318	9.93	223

**Table 6:** Measurements on the Opt2fz and Opt3 cantilever.

	Measured Quantities			Computed Quantities			
	*R* [kΩ]	*f* [kHz]	*Q*	*k* [Nm^−1^]	*F_min_* [pN]	*I_min_* [nA]	*P_min_* [fW]
Opt2fz, array1	2.16	5975	23.8	0.058	0.328	7.13	110
Opt2fz, array2	2.15	5819	25.3	0.063	0.336	7.30	115
Opt3, array1	1.74	5008	21.2	0.046	0.338	9.60	160
Opt3, array2	1.74	5205	22.3	0.038	0.294	8.35	121
